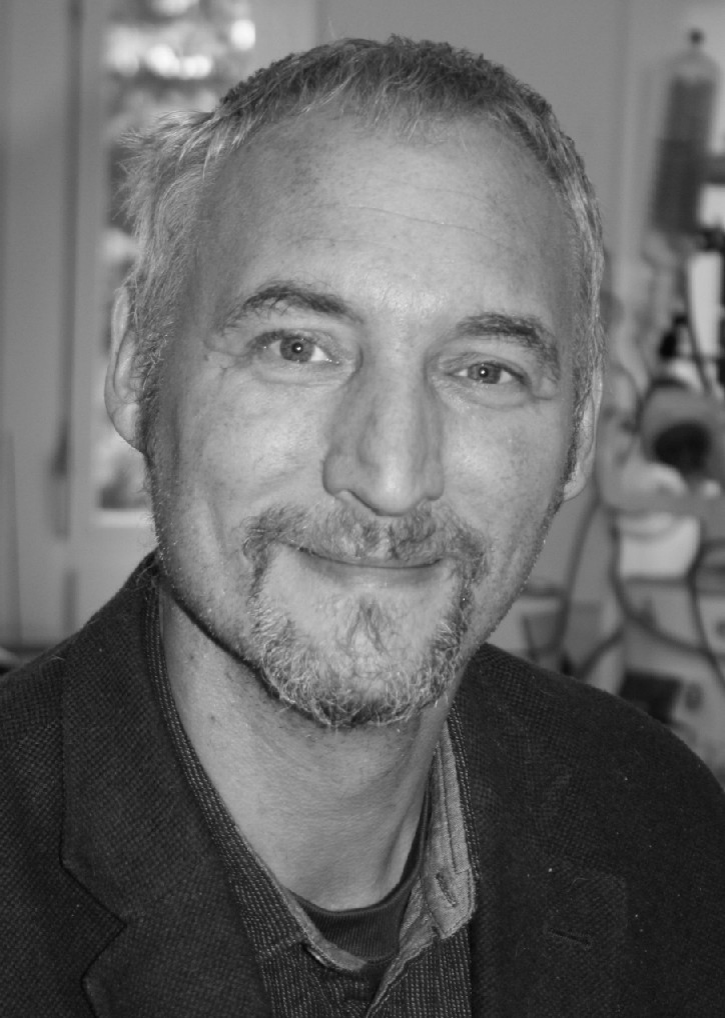# Scanning Protein Surfaces with DNA‐Encoded Libraries

**DOI:** 10.1002/cmdc.202000869

**Published:** 2020-12-28

**Authors:** Verena B. K. Kunig, Marco Potowski, Mateja Klika Škopić, Andreas Brunschweiger

**Affiliations:** ^1^ Faculty of Chemistry and Chemical Biology TU Dortmund University Otto-Hahn-Straße 6 44227 Dortmund Germany

**Keywords:** DNA-encoded libraries, drug development, peptidomimetics, protein–protein interactions, screening

## Abstract

Understanding the ligandability of a target protein, defined as the capability of a protein to bind drug‐like compounds on any site, can give important stimuli to drug‐development projects. For instance, inhibition of protein–protein interactions usually depends on the identification of protein surface binders. DNA‐encoded chemical libraries (DELs) allow scanning of protein surfaces with large chemical space. Encoded library selection screens uncovered several protein–protein interaction inhibitors and compounds binding to the surface of G protein‐coupled receptors (GPCRs) and kinases. The protein surface‐binding chemotypes from DELs are predominantly chemically modified and cyclized peptides, and functional small‐molecule peptidomimetics. Peptoid libraries and structural peptidomimetics have been less studied in the DEL field, hinting at hitherto less populated chemical space and suggesting alternative library designs. Roughly a third of bioactive molecules evolved from smaller, target‐focused libraries. They showcase the potential of encoded libraries to identify more potent molecules from weak, for example, fragment‐like, starting points.

## Graphical abstract

**DELving into the library**: Protein surface binders include chemically modified peptides and functional peptidomimetics acting as protein–protein interaction inhibitors and compounds binding to the surface of GPCRs and kinases. Several such compounds have been identified from focused libraries, thus showing the utility of DNA‐encoded chemical libraries (DELs) for improving the potency of weak, say, fragment‐like, starting points.

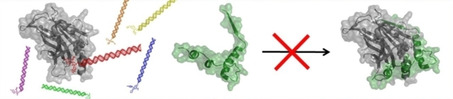

## 1 Introduction

**Figure 1:**
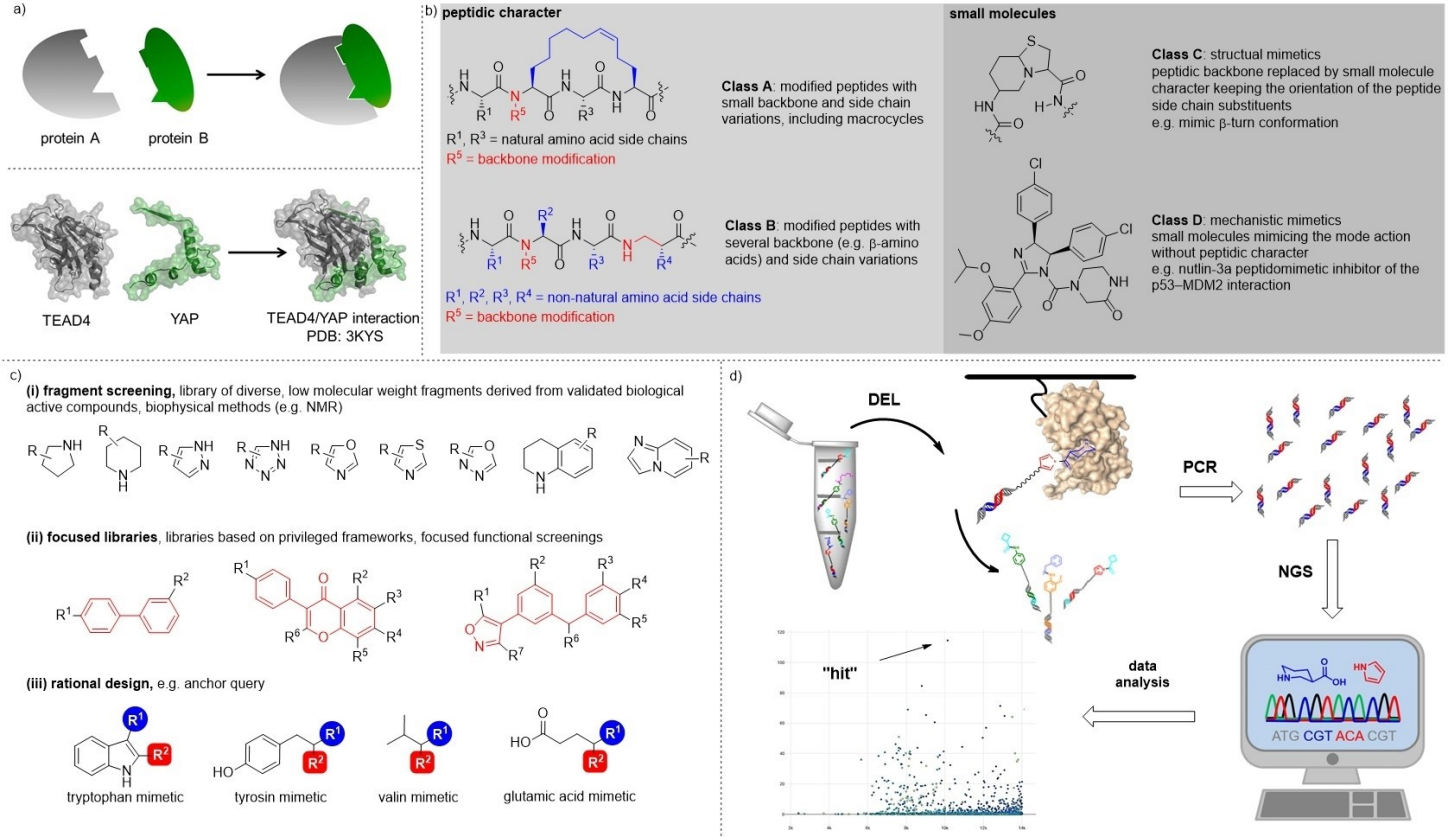
Targeting protein–protein interactions (PPIs). a) The interaction of transcription factor TEAD and the transcriptional co‐activator YAP is an exemplary protein–protein interaction. b) Classification of peptide‐derived and ‐inspired modalities for targeting PPIs. c) Design strategies for the identification of PPI inhibitors. d) Selection of DNA‐encoded libraries enables scanning protein surface with numerically large compound libraries.

Many physiological processes are regulated by direct interaction of proteins. Estimates of the size of the human interactome suggest a six‐digit number of individual protein–protein interactions (PPIs, Figure 1a), and a recently published reference map of the human interactome reported 53 000 binary protein interactions.[^1^, ^2^] These can be retrieved from databases such as HuRI, STRING, and BioGRID.[^2^, ^3^, ^4^] Several protein–protein interactions have been found to be associated with disease, providing molecular targets for intervention with therapeutic agents. A scant literature survey revealed the vast majority of well‐investigated PPIs to be associated with malignant diseases, and intense drug‐development efforts centered on this indication.[^5^, ^6^, ^7^, ^8^, ^9^] Yet, modulation of PPI holds promise for treatment of a much broader range of diseases, including devastating neurodegenerative disorders, and novel approaches to combat infectious diseases.[^10^, ^11^, ^12^, ^13^] However, a highly influential opinion piece published two decades ago cast doubt on the feasibility of inhibiting PPIs with compounds that meet the requirements for peroral application and uptake into cells for cytosolic or nuclear target engagement.^[14]^ The large binding interface, the occurrence of non‐contiguous binding areas, the shallow surface of proteins involved in PPI,^[15]^ and not the least the lack of starting points for rational drug design are formidable obstacles for inhibitor development (exemplified by the TEAD/YAP interaction, Figure 1a).[^16^, ^17^, ^18^, ^19^, ^20^, ^21^, ^22^, ^23^, ^24^] Yet, a number of clinical stage PPI inhibitors (PPIi) and approved drugs such as the BcL‐xL inhibitor Navitoclax show that this does not hold true to all disease‐relevant PPIs.^[16]^

Current drug development efforts in this field are supported by a deeper understanding of PPIs at the molecular level. Large‐scale analysis of alanine mutants revealed that in many cases protein–protein interactions critically depend on a few amino acid side chain interactions covering only a small area of the whole interface.[^25^, ^26^, ^27^, ^28^, ^29^, ^30^, ^31^, ^32^, ^33^, ^34^] Such areas are called “hot spots”, and they have been shown to be productive entry points for inhibitor design.[^34^, ^35^, ^36^] Further large scale structural analyses of proteins involved in PPIs pointed out that many of them contain cavities which could serve as locks for small organic molecule keys.[^37^, ^38^, ^39^, ^40^] The properties of these cavities were found to differ from those formed by protein targets belonging to the “druggable genome”,^[37]^ that is, certain receptor and enzyme superfamilies such as the kinase and G protein‐coupled receptor families. Likely, these cavities require chemical matter for binder/inhibitor development which is different from many of the small‐molecule designs that evolved over decades of drug research on the “druggable genome”.^[14]^

Today, the medicinal chemist has a diverse toolbox of chemical modalities and technologies available for protein binder identification, and PPIi development.^[41]^ The modalities have been classified by Grossmann et al. They range from peptides and chemically modified, for example, cyclized peptides (class A), via alternative peptide‐like oligomers such as peptoids (class B), to small molecules that are structural mimics of peptides (class C), and small molecules that mimic peptide functions (class D, Figure 1b).^[42]^ We wish to introduce here one further class of peptidomimetics, a class E that describes small‐molecule mimetics of post‐translationally modified peptides. For instance, orthosteric bromodomain inhibitors fall into this class. Important technologies for small‐molecule protein binder identification at the disposal of medicinal chemists include structure‐based (peptidomimetic) compound design exemplified by the *AnchorQuery* approach,[^35^, ^36^, ^43^, ^44^] screening of fragment libraries by biophysical and spectroscopic methods,^[45]^ the design of target‐focused screening libraries (Figure 1c),[^46^, ^47^] and, subject of this review, scanning protein surface with chemically synthesized DNA‐encoded combinatorial libraries (Figure 1d). In this review, we will summarize encoded library technologies, encoded library designs, and describe successful identification of PPI inhibitors from encoded libraries. Beyond PPI targets, we will also show protease inhibitors, as these enzymes share with PPI targets the central feature of an extended binding surface, and compounds that revealed allosteric binding sites on the surface of GPCRs and kinases, that is, “druggable genome” targets. Throughout the review, we will point out where the aforementioned technologies and encoded library technology were used in a synergistic manner for encoded library design, and DEL screening hit elaboration.

## 2 Encoded Libraries

**Figure 2:**
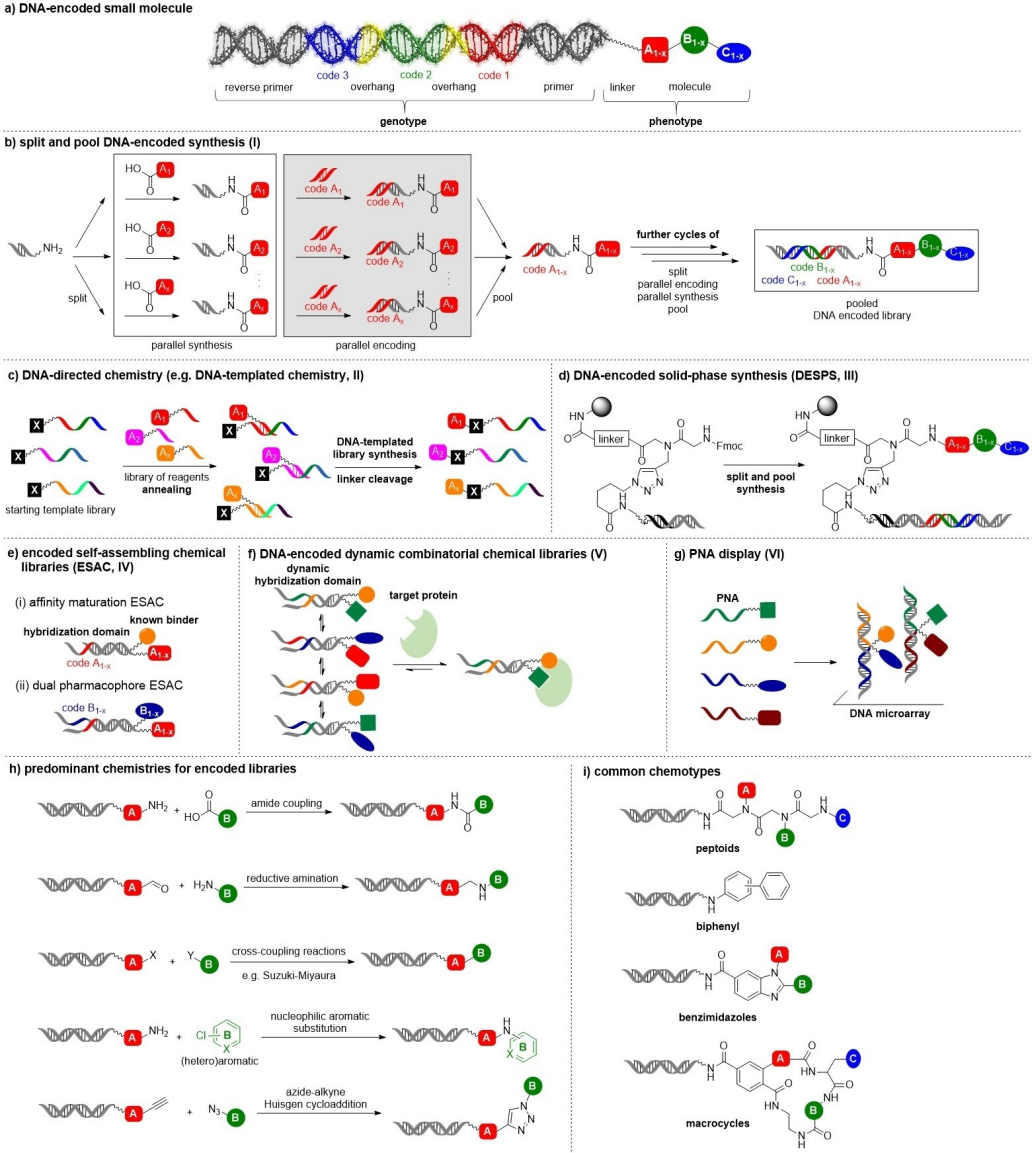
Encoded library technology. a) A DNA‐encoded small molecule. b) Split‐and‐pool DNA‐encoded library synthesis. c) DNA‐directed chemistry. d) DNA‐encoded solid‐phase synthesis. e) Encoded self‐assembling chemical libraries. f) DNA‐encoded dynamic combinatorial chemical libraries. g) PNA display. h) Prevailing reactions for encoded library design. i) Common chemotypes found in DNA‐encoded libraries.

DNA‐encoded libraries, typically abbreviated DELs, are a technology for target‐based screening that relies on phenotype‐genotype coupling (Figure 2a).[^48^, ^49^, ^50^, ^51^, ^52^] It is related to display technologies such as phage and RNA display, but uses organic preparative chemistry for the combinatorial synthesis of large numbers of encoded molecules.^[53]^ For identification of bioactive molecules, encoded libraries are typically selected on tagged recombinant proteins immobilized on a surface (Figure 1d), though alternative selection formats in solution or in cells involving covalent capture of compounds or enzymatic steps have been shown.^[52]^ Encoded one‐bead/one‐compound libraries even offer the opportunity to perform functional screens in miniaturized assays.^[52]^ Over the last three decades several encoded library formats have been introduced that shall be summarized below.

### 2.1 DNA‐encoded solution phase combinatorial chemistry (I)

The currently most common format for DEL synthesis is the solution phase split‐and‐pool approach introduced by Neri and Morgan (Figure 2b).[^54^, ^55^] This approach relies on concatenating short DNA oligomers containing genetic information for the chemical building blocks that are coupled to build up a DEL in a way that records library synthesis history. In the first synthesis cycle, a short, linker‐modified single‐stranded,^[54]^ or, as adopted by most users in the industry,^[55]^ a duplex DNA called “headpiece” is split and a first set of building blocks are coupled to the “headpiece” DNA followed by the ligation of the corresponding DNA codes. Afterwards, all products are pooled into a single vessel, and split for the next cycle of encoding and synthesis. Exponential library growth over 2–4 cycles and massive parallelization at each synthesis step led to numerically large encoded libraries.

### 2.2 DNA‐templated/directed/routed chemistry (II)

DNA‐directed approaches make use of DNA strands as barcodes for compound identification, for forcing reactants into proximity, and/or use them to program an encoded library synthesis. The group of David R. Liu exploited the barcoding and templating properties of DNA to introduce the DNA‐templated chemical libraries (DTL, Figure 2c).[^56^, ^57^] Here, the first building block is coupled to a long single‐stranded template DNA that contains coding regions for programmed library synthesis. Hybridization of the template with anticodon‐building block conjugates, chemical reaction of the building blocks followed by cleavage of the anticodon‐building block linker leads to encoded libraries. The synthesis of diverse macrocycle libraries is an impressive application of DNA‐templated chemistry.[^56^, ^57^] A related approach, called “yoctoreactor”, was developed by Hansen and co‐workers to synthesize encoded small‐molecule libraries from a DNA‐conjugated/encoded starting point for DEL synthesis and DNA‐constructs consisting of partially complementary sequences that encode bifunctional starting materials linked by a cleavable linker.^[58]^ In an approach called “DNA‐routing”, Harbury used DNA anticodon strands to direct DNA‐encoded libraries to vessels for programmed library synthesis.^[59]^

### 2.3 DNA‐encoded solid‐phase synthesis (DESPS, III)

Encoded solid‐phase chemistry offers advantages such as free choice of the solvent,[^53^, ^60^] and, as mentioned above, the perspective to employ different screening technologies for compound identification.^[52]^ The Paegel and Kodadek groups established novel approaches to DNA‐encoded one‐bead‐one‐compound (OBOC, Figure 2d) libraries.[^61^, ^62^] They modified TentaGel Rink‐amide resin with an alkyne/amine bifunctional linker, coupled the headpiece DNA by copper(I)‐catalyzed alkyne–azide cycloaddition (CuAAC) reaction to the alkyne and started encoded compound synthesis from the amine position. Unlike solution phase DELs, the DNA barcode of OBOC libraries encodes multiple copies of an encoded compound.^[61]^ Encoded OBOC libraries can either be screened by FACS to detect binding of labeled proteins to individual beads or in functional assays.[^63^, ^64^] In the latter case, molecules are removed from the solid phase and a functional read‐out is coupled to sequencing of the barcode of the active molecule.

### 2.4 Encoded self‐assembled chemical (ESAC) libraries (IV)

Neri et al. introduced a DNA‐encoded approach for fragment screening termed encoded self‐assembled chemical (ESAC, Figure 2e) libraries to identify novel ligands for macromolecular targets or for affinity maturation of known protein binders. The ESAC strategy is based on the noncovalent combinatorial assembly of complementary DNA sequences from different sublibraries. The sublibraries consist of DNA oligonucleotides containing a hybridization domain and a unique DNA barcode identifying the chemical building blocks covalently attached to the 5’ or the 3’‐end. Here, the combinatorial hybridization of relatively small sublibraries can lead to the formation of very large ESAC libraries.[^65^, ^66^]

### 2.5 Encoded dynamic combinatorial chemistry (V)

Dynamic combinatorial chemistry (DCC) refers to the combination of molecular building blocks through reversible reactions under thermodynamic control for the synthesis of complex small‐molecule libraries. An external stimulus such as adding a biomolecule can alter the thermodynamic equilibrium of the library composition.^[67]^ The utility of DCC to identify small‐molecule binders of target proteins was hampered by the lack of methodologies to analyze very complex small‐molecule mixtures. DNA‐barcoding of reactive fragments enables increasing library sizes. Encoded dynamic combinatorial chemical libraries (Figure 2f) make use of DNA‐mediated hybridization of relatively “unstable” duplex DNA oligonucleotides that can be re‐paired upon target addition to enrich high affinity fragment combinations.[^68^, ^69^] Freezing the thermodynamic equilibrium was facilitated, for example, by photo‐crosslinking or ligation of DNA oligonucleotides.[^70^, ^71^]

### 2.6 PNA display (VI)

Winssinger and co‐workers exploited the chemically much more stable peptide nucleic acid (PNA) to encode small molecules (Figure 2g). Synthesizing a PNA‐encoded compound library benefits from the opportunity that the PNA tag can be co‐synthesized with the organic molecule by traditional solid‐phase synthesis strategy. One limitation of using PNA is that it cannot function as a template for amplification and sequencing using polymerases. Instead, DNA arrays were used to display PNA‐tagged small molecules, for example, for fragment screening.[^72^, ^73^]

### 2.7 Encoded library chemical space

The reactions for encoded library design have been extensively reviewed elsewhere.[^74^, ^75^] DNA‐encoded compounds mirror the linear process of DNA barcode concatenation. As the barcode grows in a linear manner, barcoded compounds are concatenated from building blocks, either in linear fashion, or coupled successively to a central scaffold displaying functional groups. Linear structures may be cyclized in the final step, yielding macrocyclic structures. Published screening hits from DEL screens have for instance validated carbonyl chemistries, C−C cross‐coupling reactions, CuAAC “reaction, nucleophilic aromatic substitution reactions, and benzimidazole synthesis for library construction (Figure 2h). These reactions enrich sp^2^‐rich molecules and structures with peptidic character in screening libraries (Figure 2i).

## 3 Inhibition of Protein–Protein Interactions

### 3.1 Chemically modified peptides and peptide macrocycles

**Figure 3:**
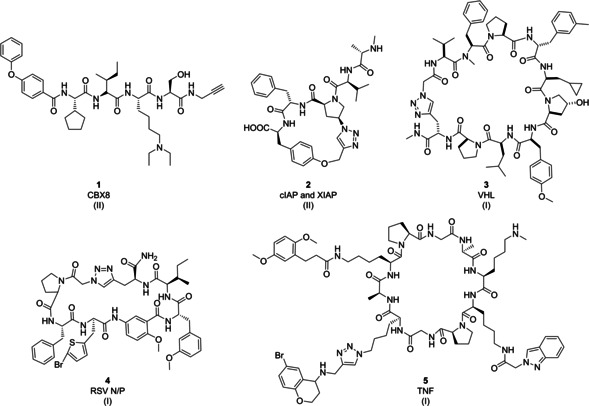
Examples of chemically modified peptides and peptide macrocycles as PPI inhibitors (class A peptidomimetics). Roman numerals in brackets indicate the DEL technology origin of the compound.

*CBX8*: Polycomb group (PcG) proteins are transcriptional repressors^[76]^ that are part of polycomb repressive complex 1 (PRC1) and polycomb repressive complex 2 (PRC2).[^77^, ^78^, ^79^] CBX8 has recently emerged as a potential drug target in a variety of malignancies, such as leukemia with MLL (mixed lineage leukemia) translocations.^[80]^ The groups of Krusemark and Dykhuizen employed a DNA‐routing approach to identify potent and selective CBX8 chromodomain (ChD) inhibitors.[^81^, ^82^] Selection of a peptide‐DEL against a panel of CBX ChDs led to the identification of peptide sequences with increased affinity and selectivity to CBX8 over CBX7 ChD. The authors showed then the utility of encoded combinatorial libraries by designing a focused encoded library with the aim of improving inhibitor affinity, selectivity, and cell permeability.^[82]^ Several molecules were selected for off‐DNA experiments. The *K*_d_ of fluorescently labeled compound **1** for the CBX8 ChD was ∼800 nM which was similar to ∼500 nM value obtained with unlabeled **1** (Figure 3) in a thermal shift assay. This compound showed high selectivity for CBX8 over CBX4 and CBX6, 20‐fold selectivity over CBX7, and fivefold selectivity over CBX2. NMR spectroscopy studies indicated that inhibitor **1** can compete with histone tail binding. Biotinylated compound **1** was used to enrich CBX8 and other paralogs from mouse embryonic fibroblast (MEF) lysates and HEK293T lysates. Chromatin immunoprecipitation (ChIP) followed by quantitative PCR (ChIP‐qPCR), and sequential salt extraction (SSE) validated the ability of **1** to disrupt CBX8 association with chromatin.

Compound **1** demonstrated antiproliferative activity in CBX8‐dependent leukemia cells with MLL‐AF9 translocations (THP1 cells), with IC_50_ of 26 μM. In addition, inhibition of CBX8 ChD with **1** decreased the transcription of MLL‐AF9 target genes (HOXA9, CDK6, MYB, RUNX2, and RUNX3) in THAP1 cells after 2 days of the treatment. Overall, this study highlighted the potential of small, focused DELs for targeting challenging proteins.^[82]^

*cIAP and XIAP*: Inability of cells to execute apoptosis, or programmed cell death (PCD), is associated with many malignant diseases.^[83]^ Cellular inhibitor of apoptosis proteins (cIAPs) inhibit the extrinsic pathway of PCD by blocking the activated initiator caspase‐8 protein, while X‐chromosome‐linked inhibitor of apoptosis proteins (XIAP) directly binds and inhibits both initiator and effector caspases associated with both PCD pathways. A 160 000‐member DNA‐templated library of macrocyclic pentapeptides was designed around the N‐terminal alanine‐residue of the native XIAP binding peptide sequence AVPI and screened for the identification of potent cIAP/XIAP antagonists.^[84]^ The DNA‐programmed peptide library was synthesized through five coupling steps with 20 different natural and unnatural amino acids and a final cyclization step via CuAAC reaction. Initial hits showed activity in the micromolar range, and their amino acid sequence was similar to the N‐terminal sequence of Smac. In follow‐up studies, synthesis of a small focused 1760 member DEL and structure‐guided compound optimization led to compound **2** (Figure 3) which demonstrated good balanced affinity for XIAP BIR2 (IC_50_=0.14 μM), XIAP BIR3 (IC_50_=0.16 μM), and cIAP1 BIR3 (IC_50_=0.02 μM). Interestingly, a dimeric molecule that formed as a side‐product, showed nanomolar affinity and was used for further inhibitor design cycles.^[84]^

*VHL*: Encoded split‐and‐pool peptide chemistry with a final macrocyclization step by CuAAC reaction yielded a library of macrocyclic peptides through six reaction cycles leading to 2.4×10^12^ peptides of variable ring sizes ranging from four to 20 amino acids.^[85]^ Library synthesis involved coupling of natural and non‐natural amino acids, dipeptides, and tripeptides. The authors of this work included hydroxyproline, a ligand of the E3 ubiquitin ligase Von‐Hippel‐Lindau tumor suppressor (VHL), in the library.^[86]^ This E3 ubiquitin ligase is involved in the ubiquitination and subsequent degradation of a hypoxia‐inducible factor (HIF). HIF is a transcription factor with a critical role in the regulation of gene expression by oxygen. The selection against VHL validated the peptide macrocycle DEL, enriching macrocycles with hydroxyproline residues such as compound **3** (Figure 3). These compounds could be used as tools to probe the VHL/HIF protein–protein interaction.^[85]^

*RSV N‐protein/P‐protein*: The same encoded macrocycle library was selected against respiratory syncytial virus (RSV) N‐protein. Interaction between RSV N‐protein and P‐protein is crucially important for the replication of RSV,^[87]^ and inhibitors of this PPI hold promise for the treatment of RSV infections.^[88]^ Macrocyclic peptides with good predicted permeability and solubility, and with at least 10‐fold enrichment compared to the corresponding linear peptides were selected for functional studies. Their binding was confirmed by an affinity selection–mass spectrometry (AS‐MS) assay. Functional activity of macrocyclic peptide **4** (Figure 3) was demonstrated in a time‐resolved fluorescence resonance energy transfer (TR‐FRET) assay that detected disruption of the interaction between RSV N‐protein and P‐protein (IC_50_∼100 nM). Comparison with linear peptides showed that peptide macrocyclization had a positive effect on affinity for the RSV N‐protein.^[85]^

*TNF*: A split‐and‐pool DEL was designed by Neri and co‐workers to mimic antibody–antigen recognition through three diversity elements displayed on a structurally defined macrocyclic scaffold.^[89]^ A previously reported macrocyclic scaffold with antiparallel β‐sheets[^90^, ^91^, ^92^] was selected as a platform for the library synthesis. This scaffold was substituted on three orthogonally protected amines by combinatorial amide coupling reactions and CuAAC leading to 35 million encoded macrocycles. The library was validated by selection against carbonic anhydrase IX (CAIX), horseradish peroxidase (HRP), and tankyrase 1 (TNKS 1). In addition to the identification of novel inhibitors of human serum albumin (HSA), alpha‐1 acid glycoprotein (AGP), calmodulin (CaM), and prostate‐specific antigen (PSA), screening on tumor necrosis factor (TNF) also led to the identification of TNF inhibitor **5** (Figure 3). Compound **5** demonstrated activity against recombinant TNF (*K*_d_=15 μM) and TNF‐antibody fusion L19‐TNF (*K*_d_=6.1 μM). This study demonstrated that large libraries consisting of side‐chain diversity on a constant macrocycle scaffold can deliver valuable protein binders. In addition, the structure–activity relationships (SARs) that were recognized after selection experiments suggested that design of second‐generation libraries around the enriched members might lead to further potency gains.^[89]^

### 3.2 Peptoids

**Figure 4:**
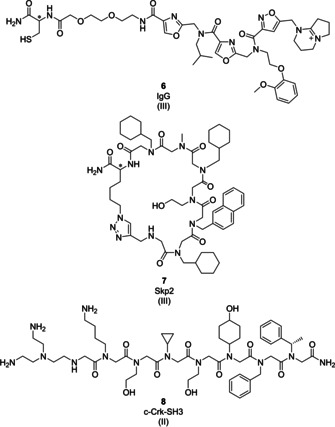
Examples of peptoids as PPI inhibitors (class B peptidomimetics). Roman numerals in brackets indicate the DEL technology origin of the compound.

*IgG*: Distinguishing the latent infectious condition (LTB) from active infectious condition (ATB) during *Mycobacterium tuberculosis* (Mtb) infection is important for a proper interpretation of the patient‘s health state.^[93]^ Therefore, the development of highly sensitive and specific diagnostic tools is very important. The OBOC DEL technology was combined with FACS‐based screening to discover ATB‐specific serum ligands that bind IgG.^[94]^ A 448 000‐member OBOC DEL library was screened against LTB and ATB serum pools using fluorescently labeled IgG. The library displayed diversity at three positions and it was synthesized by peptide coupling in combination with a peptoid construction via use of halogenated acids and amines for halide substitution. Competition binding data revealed four ligands that maximally sampled the ATB patient serum samples with ligand **6** (Figure 4) as a potent and selective ATB serum IgG binder that mimics a native Ag85B epitope. The sequencing data suggested that conformational constraint is important for IgG binding.^[94]^

*Skp2*: Selection of a DNA‐encoded OBOC library of cyclic peptoids against oncogenic protein Skp2 (S‐phase kinase‐associated protein 2) identified the hit compound **7** (Figure 4, *K*_d_=7.51 μM).^[95]^ Synthesis of the peptoid library was initiated on the amino‐modified TentaGel beads. First, the Fmoc‐Ahx‐OH linker was coupled. Following Fmoc‐deprotection, a photocleavable ANP linker and an azido‐modified amino acid were coupled. The amino group of the linker was reacted with chloroacetic acid, and the resulting chloride was substituted with 15 different amine monomers (aliphatic, aromatic, heteroaromatic). The reaction sequence of chloroacylation and nucleophilic substitution with amines was repeated for the synthesis of a combinatorial hexamer peptoid library of around 11 million macrocycles. The OBOC DEL was finalized with a CuAAC reaction for the macrocycle formation. To reduce the number of initial hits identified from the affinity‐based DEL selection against the Skp2‐Skp1 complex for off‐DNA resynthesis and validation, and in particular to avoid resynthesis of false‐positive binders arising from DNA tag interaction with the target, the authors decided to synthesize a focused off‐DNA sublibrary of cyclic peptoids on bilayer beads and screened it against Skp2. Based on the screening results, only five molecules were selected for off‐DNA hit validation. In this research work, the hit identification omitted the next‐generation sequencing (NGS) but took the advantage of T‐vector (TA) cloning of the selected and PCR‐amplified DNA.^[95]^

*N‐Crk‐SH3*: The c‐Crk protein, a Src homology 3 (SH3) domain containing adaptor protein, plays an important role in signal transduction. Its dysregulation is associated with malignant diseases.^[96]^ SH3 domains are involved in numerous PPIs^[97]^ and are considered promising drug targets.[^98^, ^99^] Selection of a 100 million‐member octamer peptoid PNA‐encoded library against the N‐terminal SH3 domain of c‐Crk protein (N‐Crk‐SH3) led to the identification of ten peptoids selected for off‐DNA validation.^[100]^

This library was synthesized by a reaction sequence of chloroacylation and nucleophilic substitution with amines which was repeated until octamer construction. The peptoids shared some structural features such as the tris‐(2‐amino‐ethyl)amine side chain at the N‐terminal end, bulky side chains at the first and/or second position, and three or four small side chains at the central peptoid portion. Six ligands from different ligand families showed binding affinities (*K*_d_=10–100 μM) for N‐Crk‐SH3, similar to those of natural SH3 peptide‐based binders^[101]^ with peptoid **8** (Figure 4) exhibiting the highest affinity for N‐CrkSH3 (*K*_d_=16 μM).^[100]^

### 3.3 Structural small‐molecule peptidomimetics

**Figure 5:**
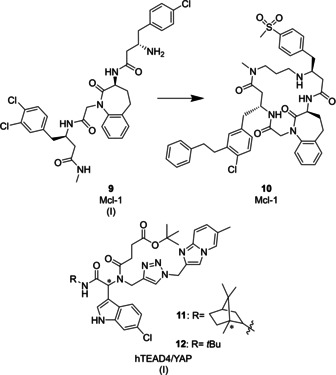
Examples of structural small‐molecule peptidomimetics as PPI inhibitors (class C peptidomimetics). Roman numerals in brackets indicate the DEL technology origin of the compound.

*Mcl‐1*: Mcl‐1 is an antiapoptotic protein from the Bcl‐2 protein family. Identification of small‐molecule mimetics of the BH3 domain of the pro‐apoptotic inducer protein NOXA is of great interest as these molecules could potentially bind to Mcl‐1 and disrupt protein–protein interaction between Mcl‐1 and effector proteins BAX or BAK to initiate apoptosis.[^102^, ^103^] A drug discovery program aimed at identifying novel Mcl‐1 inhibitors, employed affinity‐based screening of a tripeptide DEL against Mcl‐1.^[104]^ The co‐crystal structure of hit compound **9** (Figure 5, IC_50_=2 μM), which comprises a dihydrobenzazepine as a β‐turn mimetic core, and Mcl‐1 showed that **9** binds to the BH3 binding groove of Mcl‐1 where it accommodates a β‐turn conformation. Structure‐guided rigidification of the compound toward the bound state through macrocyclization **10** improved the potency of the initial hit by nearly three orders of magnitude (IC_50_=<3 nM).^[104]^

*hTEAD4/YAP*: Dysregulation of protein–protein interactions between transcriptional enhancer factor‐1 domains (TEAD1‐4) and co‐transcription factor Yes‐associated protein (YAP), late Hippo signaling pathway effectors, is associated with oncogenic mechanisms.[^105^, ^106^, ^107^] Recently, screening of a small thymidine‐initiated DEL (tiDEL) of peptidomimetics against YAP‐interacting domain of human TEAD4 (hTEAD4) led to the identification of two PPI inhibitors **11** (Figure 5, IC_50_=6.75 μM) and **12** (IC_50_=5.65 μM).^[108]^ This library was synthesized around the tryptophan side chain as an “anchor motif” with indoles introduced into the library via Ugi four‐component reaction as tryptophan mimetics and CuAAC for library diversification. PPI inhibitors **11** and **12** exhibited different binding modes which are still under investigation. Both compounds showed hTEAD4/YAP interaction inhibition, however, only compound **11** exhibited inhibition of the palmitic acid‐hTEAD4 interaction that takes place in the so called “central pocket” of hTEAD. Notably, compound **11** demonstrated perturbation of the expression of CTGF gene which is under control of these Hippo pathway effectors.^[108]^

### 3.4 Functional small‐molecule peptidomimetics

**Figure 6:**
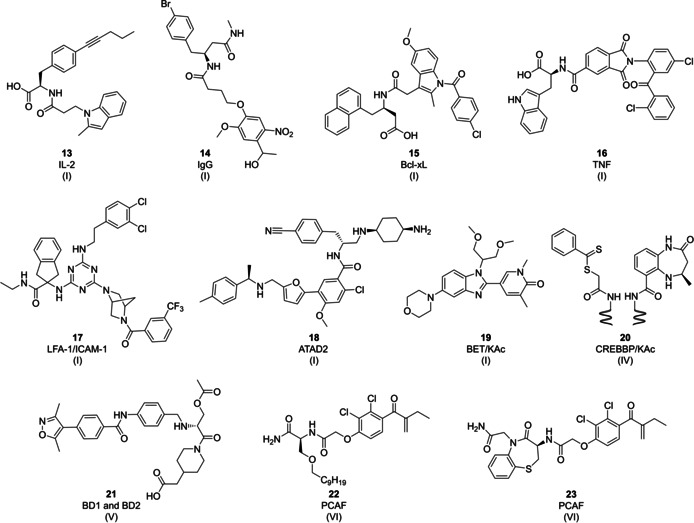
Examples of functional small‐molecule peptidomimetics and functional mimetics of post‐translationally modified peptide sequences as PPI inhibitors (class D and E peptidomimetics, see text for distinction). Roman numerals in brackets indicate the DEL technology origin of the compound.

*IL‐2*: Interleukin‐2 (IL‐2) is a pro‐inflammatory cytokine which is involved in high‐affinity protein–protein interaction with its cognate receptor. In an early DEL project, a 30 000‐member split‐and‐pool DNA‐encoded library synthesized by two combinatorial amide coupling reactions was screened against a panel of proteins.^[109]^ Selection against human IL‐2 revealed that many enriched sequences were coding for 2‐methyl‐1*H*‐indole derivatives. Investigation of their binding mode in molecular docking studies revealed that the indole moiety is likely pointing towards the IL‐2 surface. The most potent compound identified from the library was IL‐2 inhibitor **13** (Figure 6, *K*_d_=2.5 μM) which demonstrated selective IL‐2 inhibition in a T‐cell proliferation assay.^[109]^

*IgG*: Polyclonal human IgG binders are of great interest because they can be used to develop affinity beads for purification of monoclonal antibodies. Screening of 4 000 encoded amides revealed a number of small‐molecule binders of polyclonal human IgG with **14** (Figure 6) as the most enriched compound.^[54]^

*Bcl‐xL*: Selection of the same library on Bcl‐xL led to the identification of **15** (Figure 6) as an inhibitor of the antiapoptotic protein Bcl‐xL, which is an attractive target for the development of antitumor drugs. A fluorescein‐labeled derivative of compound **15** showed a higher affinity (*K*_d_=0.93 μM) for Bcl‐xL than the parent molecule probably due to additional interactions of the dye with the target protein.^[110]^

**TNF** Selection of a small 4 000‐member DEL, synthesized by Diels‐Alder cycloaddition of 20 DNA‐tagged dienes with 200 maleimides, on tumor necrosis factor protein (TNF) delivered hit molecule **16** (Figure 6) that was validated by fluorescence polarization assay against the trimeric EDB‐TNF fusion protein (*K*_d_=15 μM). This study was one of the early demonstrations of the applicability of DEL technology for the *de novo* discovery of protein–protein interaction inhibitors.^[111]^

### 3.5 Allosteric mode of action

*LFA‐1/ICAM‐1*: The integrin LFA‐1 (lymphocyte function‐associated antigen‐1) is a leukocyte cell adhesion molecule binding to its major ligand ICAM‐1 (intercellular adhesion molecule‐1) on endothelial and dendritic cells.^[112]^ Because of its key role in regulating leukocyte trafficking during inflammation and in inducing immune responses, it represents an established therapeutic target for the treatment of autoimmune and inflammatory diseases.[^113^, ^114^] DEL technology was utilized for the identification of small‐molecule inhibitors of LFA‐1/ICAM‐1 PPI.^[115]^ The DEL was synthesized according to a seminal publication from Barry Morgan et al. by four reaction cycles yielding 4.1 billion 1,3,5‐triaminotriazines.^[55]^ Affinity selection was performed in three rounds against the soluble streptavidin‐tagged LFA‐1 I domain. Compound **17** (Figure 6) showed the inhibition of LFA‐1/ICAM‐1 interaction in the ELISA‐type ligand binding assay (IC_50_=23 nM) and demonstrated inhibition of cell adhesion to ICAM‐1 in a human lymphocyte Jurkat cell‐line that expresses native WT LFA‐1. It exhibited the same mode of action as an established allosteric antagonist LFA703, and likely binds to the allosteric pocket below the C‐terminal helix in the I domain. Finally, it was demonstrated that **17** selected on the soluble LFA‐1 I domain retained affinity for native LFA‐1 expressed on the cell membrane.^[115]^

*ATAD2*: ATPase family AAA‐domain containing protein 2 (ATAD2 or ANCCA) is a bromodomain (BD) containing protein which acts as an epigenetic regulator and transcriptional cofactor for oncogenic transcription factors, such as ERα, AR, E2F, and Myc.[^116^, ^117^, ^118^] ATAD2 interacts with histone acetylation marks on newly synthesized histone H4.^[119]^ Its overexpression is associated with different cancer types, however, its validation as a drug target is very challenging due to the lack of isoform‐selective and cell active ATAD2 inhibitors. Screening a pool of 11 DELs consisting of 65 billion compounds against ATAD2 led to the identification of an isoform‐selective inhibitor derived from a sublibrary.^[120]^ This sublibrary was synthesized from three sets of building blocks: 300 different amino acids were used as the first set of building blocks (BBs), 150 different formyl acids were introduced by acylation as the second set of BBs, the third set of BBs consisted of 2341 amines introduced by reductive amination. Hit‐to‐lead optimization led to a selective ATAD2 inhibitor, compound **18** (Figure 6, BAY‐850) which displaced the tetra‐acetylated histone H4 peptide in orthogonal binding competition assays (IC_50_=157 nM) and demonstrated activity in cells. Interestingly, compound **18** showed an unusual mode of action as it induced protein dimer formation.^[120]^

### 3.6 Functional small‐molecule mimetics of post‐translationally modified peptides

*BET/KAc*: The bromodomain and extraterminal domain (BET) family of bromodomain containing proteins (BCPs) are epigenetic readers that recognize N‐acetyl lysine (KAc) modifications on histone proteins. Dysregulation of these interactions is associated with diseases, small‐molecule inhibitors of the BET/KAc interaction have entered clinical trials.^[121]^

A DEL screening campaign yielded a 2‐(4‐hydroxy‐3,5‐dimethylphenyl)benzimidazole series from a 117 million‐member benzimidazole library.^[122]^ The library was synthesized via three reaction cycles. First, a DNA‐conjugated 4‐fluoro‐3‐nitro benzoate was reacted by nucleophilic aromatic substitution with 65 monoprotected diamines. Following nitro reduction, the diaminoaryls were condensed with 922 aldehydes to DNA‐tagged benzimidazoles. Following the amine deprotection, the library was substituted with 1960 amine capping groups. The drug discovery program progressed by hybridizing the DEL hit with an *N*‐methylpyridone fragment, a KAc mimetic obtained from fragment‐based screening, demonstrating a successful combination of DEL technology with fragment screening for the development of the advanced lead molecule I‐BET469 **19** (Figure 6, BRD4 BD1 IC_50_∼10 nM).^[122]^

*CREBBP/KAc*: The cyclic‐AMP response element binding protein (CREB) binding protein (CREBBP) is a transcription factor which is involved in more than 400 protein–protein interactions.^[123]^ The CREBBP bromodomain displays two neighboring binding pockets: the KAc binding pocket and the induced‐fit pocket. An encoded self‐assembly chemical library was employed to identify fragments that can bind synergistically to the two adjacent CREBBP bromodomain binding sites.^[124]^ The focused ESAC library was assembled by hybridization of partially complementary 5’‐amino‐modified oligonucleotides linked to 787 fragments (sublibrary A) and 3’‐amino‐modified oligonucleotides linked to 424 fragments (sublibrary B). The library incorporated 4,5‐dihydrobenzodiazepinone (*R*)‐THBD‐based ligand, a known binder of the KAc‐binding pocket.^[125]^ Affinity‐based selections identified a dual fragment combination **20** (Figure 6, *K*_d_=0.86 μM) that exhibited 30‐fold higher affinity for the CREBBP bromodomain than (*R*)‐THBD paired with an acetyl moiety.^[124]^

*BD1 and BD2*: Selection of a focused DNA‐encoded dynamic library (DEDL) against bromodomain 4 (BRD4) revealed BD1 and BD2 inhibitor **21** (Figure 6).^[126]^ In general, design of this DEDL requires a known protein binder which can be utilized as an “anchor” with an aldehyde group and DNA‐encoded library whose members are displaying a primary amine. DNA‐encoded compounds compete for the anchor via imine formation, the protein target orchestrates the formation of high‐affinity binders, and finally reductive amination terminates the dynamic exchange. Three BD1/BD2 anchors, known binders with different affinities for BRD4, were mixed each with 67 600 encoded dipeptides to form dynamic libraries that were screened against BD1 and BD2 in solution. The selection results showed that most of the compounds were more active than the anchors themselves, making this method useful for ligand optimization across a wide range of binding affinities. For an isoxazole based anchor with an IC_50_ in the mid‐micromolar range, the IC_50_ improved in the case of hybrid **21** 26‐fold to 1.55 μM for BD1 and 29‐fold to 1.46 μM for BD2.^[126]^

*PCAF*: Selection of a library of PNA‐encoded small‐molecule fragments against P300‐CBP associated factor (PCAF) led to the identification of 25 fragments that were used as a starting point for the synthesis of a focused PNA‐encoded library of 625 compounds displayed on DNA microarray.^[127]^ This small library was synthesized with two sets of building blocks. As the first set of building blocks, 25 different natural and unnatural amino acids were introduced, and then different carboxylic acids, sulfonyl chlorides, and alkynes were introduced as the second set of building blocks by acylation, sulfonylation, and CuAAC reaction, respectively. This small library yielded two PCAF binders, **22** and **23** (Figure 6), which comprised ethacrynic acid, an FDA approved drug for the treatment of high blood pressure. Incubation of these biotin‐labeled compounds with purified PCAF and PCAF in the crude lysate resulted in PCAF labeling which showed that these ethacrynic acid derivatives were engaged in covalent interactions with PCAF cysteine residues. Both compounds were used to assess the position and reactivity of different cysteine residues of 32 tested bromodomains and showed differential reactivity with different bromodomains, for example, compound **22** reacted faster with BRD7, while compound **23** reacted faster with CREBBP. Both compounds proved useful for proteomic analysis as they were able to enrich very low concentrations of PCAF from cell lysates.^[127]^

## 4 Proteases

### 4.1 Chemically modified peptides and peptide macrocycles

**Figure 7:**
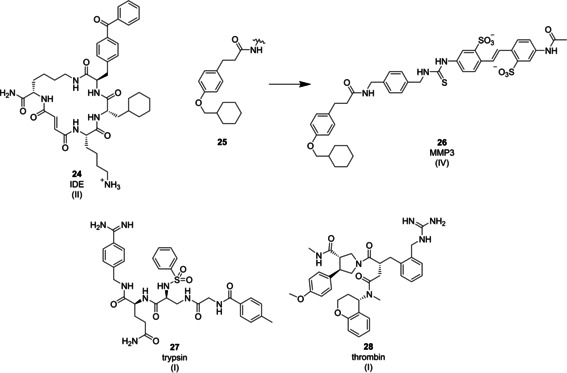
Examples of protease inhibitors (class A (**24**) and D peptidomimetics). Roman numerals in brackets indicate the DEL technology origin of the compound.

*IDE*: Insulin‐degrading enzyme (IDE) modulates blood glucose levels by degrading both blood glucose lowering insulin and blood glucose elevating glucagon. Selective inhibition of enzymatic insulin degradation versus glucagon degradation would open a novel treatment option for acquired diabetes. Liu et al. screened a small library of 13 000 peptide macrocycles on IDE and could identify a class of molecules, exemplified by **24** (Figure 7), that blocked access of insulin to the enzyme selectively and inhibited insulin degradation with an IC_50_ of 50 nM, while the ternary complex of IDE, inhibitor, and glucagon was still catalytically active. As a consequence of the unique binding mode, these molecules reprogrammed the substrate specificity of IDE.[^128^, ^129^]

### 4.2 Functional peptidomimetics

*MMP3*: Neri et al. used the ESAC technology for the identification of inhibitors of stromelysin‐1 (MMP‐3), a matrix metalloproteinase, yielding novel inhibitors with micromolar potency. Matrix metalloproteinases are zinc‐containing, extracellular endopeptidases that are involved in tissue remodeling processes; several enzymes of this family are associated with cancer and inflammatory diseases. A DNA‐encoded library with 550 compounds was selected against human MMP3 identifying a strongly enriched fragment‐like compound **25** (Figure 7) which was then used as an anchor structure for the assembly of the 550‐member ESAC sublibrary to identify more potent, bidentate inhibitors. Dual‐pharmacophore selections against MMP3 resulted in the identification of a specific pair of compounds (structure I and II) which were then combined by different linkers for the synthesis of MMP3 inhibitors binding synergistically to the target protein among which compound **26** showed the highest inhibitory potency with an IC_50_ of 9.9 μM.^[130]^

*Trypsin and thrombin*: Focused split‐and‐pool DNA‐encoded libraries were designed by the groups of Neri and Matzuk, respectively, around the oxyanion hole‐binders benzamidine and guanidine to probe the surface around the active site of the serine proteases trypsin and thrombin, respectively. Successful identification of nanomolar inhibitors of trypsin **27** (Figure 7), and thrombin **28** (Figure 7), showed the potential of combinatorial libraries to identify highly potent protease inhibitors by densely covering chemical space around weakly active starting points.[^131^, ^132^]

## 5 Peptide‐Competitive Ligands for Receptors and Enzymes

### 5.1 Chemically modified peptides and peptide macrocycles

**Figure 8:**
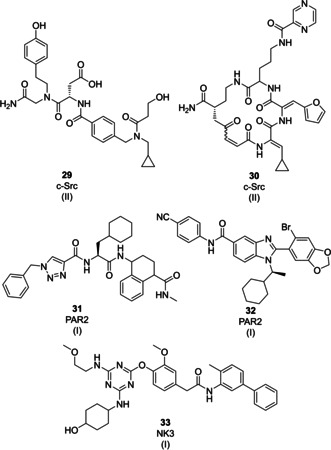
Examples of peptide‐competitive ligands for receptors and enzymes (see text for compound classification). Roman numerals in brackets indicate the DEL technology origin of the compound.

*c‐Src kinase*: Krusemark reported the synthesis of a 550 000‐member phenol containing peptidomimetic DNA‐encoded library for the identification of unnatural substrates of the tyrosine protein kinase c‐Src that can serve as artificial substrates and potentially as protein substrate competitive inhibitors. The DEL contained native peptides, non‐natural peptides and peptoid‐inspired structures. The library was treated with ATP and Src kinase followed by affinity selection assay using a non‐specific phosphotyrosine‐binding antibody for the enrichment and identification of substrate molecules of c‐Src. Substrate‐mediated selection led to the identification of a lead compound **29** (Figure 8) that was able to serve as a substrate for phosphorylation and also to promote ATP hydrolysis. Binding of this compound to the c‐Src:ATP complex was confirmed using NMR and an ester derivative of the hit compound showed inhibition of Src‐dependent signaling in NME cells.^[133]^

*c‐Src kinase*: The aforementioned macrocycle library from Liu et al. was selected on a set of proteins, among them several kinases. Compound **30** (Figure 8), inhibited c‐Src kinase activity with an IC_50_ of 960 nM and high selectivity versus a panel of 58 kinases. X‐ray structures revealed a bisubstrate mode of kinase inhibition, the macrocycle occupied the ATP‐binding pocket and blocked at the same time the substrate peptide‐binding patch, locking the kinase in an inactive conformation.[^134^, ^135^, ^136^]

### 5.2 Functional peptidomimetics

*PAR2*: Protease‐activated receptors are activated by enzymatic cleavage of an extracellular domain, unmasking the peptide sequence SLIGKV which binds to the receptor transmembrane domain. Excessive receptor activation is associated with inflammatory diseases. Researchers from X–Chem and AstraZeneca screened several large split‐and‐pool DELs that included capped diamide and benzimidazole DELs with 225 million and 7 million compounds, respectively, on a mutated, stabilized variant of the receptor in the presence and absence of a small‐molecule antagonist, revealed several classes of compounds. Functional and structural studies showed that compound **31** (Figure 8) acted as functional mimic of the PAR‐activating peptide sequence, while compound **32** bound to a previously unknown allosteric site and caused structural rearrangements of the receptor that precluded activation.[^137^, ^138^]

*NK3 receptor*: The neurokinin receptor NK3, a G protein‐coupled receptor, is activated by neuropeptides. It was used by a research team from GSK as a model system to establish DEL selection experiments on cell membranes. Selection of split and pool libraries on NK3 receptors that were overexpressed on HEK293 cells yielded several hit clusters, including compound **33** (Figure 8). Of note, the authors remarked that the hit clusters did not show similarity to established NK3 receptor antagonists, although such chemotypes were present in the library, furthermore DEL selections were productive on receptors for chemokines, peptide hormones and lipids, whereas they provided less hit matter on receptors for low‐molecular‐weight signaling molecules.^[139]^

## 6 Conclusions

**Figure 9:**
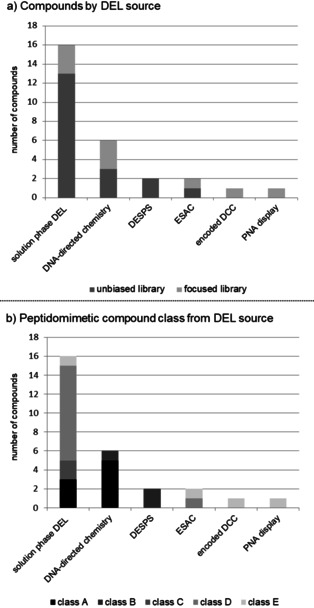
Statistical analysis of the compounds discussed in this review. a) Compounds identified from focused versus unbiased encoded library designs. b) Peptidomimetic compound classes identified from different DEL technologies.

Scanning of target protein surfaces with DNA‐encoded libraries has delivered several compounds that inhibit protein–protein interactions. More than half of these compounds (16 out of 28) originate from solution‐phase split‐and‐pool combinatorial libraries, a fact that reflects the widespread uptake of this library technology (Figure 9). Most of these molecules belong to the classes C–E of functional small‐molecule peptidomimetics, and only a minority are oligomeric, peptide compounds. DNA‐directed chemistry on the other hand accounted for six compounds, all of which belong to the oligomeric peptides and peptoids (classes A and B peptidomimetics), and also the published hits of encoded solid‐phase library screens represent bioactive peptoids. DNA‐encoded fragment screens, either performed by the ESAC technology, dynamic combinatorial chemistry, or PNA display hold much promise to scan protein surface. Yet, they have to date yielded only a handful of PPI modulators. Likely this is due to the fact, that these approaches are practiced by very few research groups. Roughly a third of the compounds shown in this review were identified from smaller, target‐focused libraries. They hint at the potential of DEL technology to improve the potency of weak starting points, for example, from fragment screening. Such approaches certainly benefit from the dense coverage of chemical space by combinatorial compound synthesis.

Surprising to us was the scarce use of structural peptide mimetics for library design. These hold much promise for the identification of PPI modulators and may either be introduced by reaction methodology as for instance multicomponent reactions or by scaffolds that mimic protein secondary structure. The design of such molecule libraries may take into account the guidelines that have been published by Kihlberg et al. and adopted in a macrocycle design by the Liu group.[^57^, ^140^] As a final remark, we wish to point out that DEL technology may provide excellent starting points for the development of protein‐degrading or other hetero‐bifunctional molecules.

## Conflict of interest

The authors declare no conflict of interest.

## Biographical Information

*Verena B. K. Kunig received her M.Sc. degree in chemical biology from the TU Dortmund in 2017. She is currently a PhD student under the supervision of PD Andreas Brunschweiger at the same institution. She is developing reaction methodology for DNA‐encoded library synthesis and assay technology for encoded library selection*.

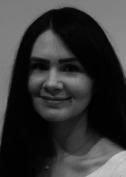

## Biographical Information

*Marco Potowski received his M.Sc. degree in chemical biology at TU Dortmund University in 2010 and his PhD under the supervision of Prof. Herbert Waldmann at the Max Planck Institute (MPI) of Molecular Physiology Dortmund in 2015. After postdoctoral research in Prof. Waldmann's group until 2017, he joined PD Andreas Brunschweiger's group at the TU Dortmund working on the development of DNA‐compatible reactions for DEL synthesis*.

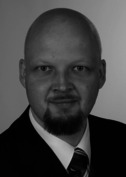

## Biographical Information

*Mateja Klika Škopić received her M.Sc. degree in chemistry from the Faculty of Chemical Engineering and Technology, University of Zagreb. After working for almost two years as a medicinal chemist in a biotech company in Zagreb, she joined the research group of PD Andreas Brunschweiger at TU Dortmund, where she earned her PhD in the field of DNA‐encoded libraries. She is currently working as a postdoctoral researcher in the Brunschweiger lab on the development of micellar catalysis for DEL synthesis*.

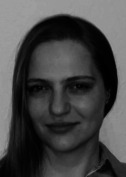

## Biographical Information

*PD Andreas Brunschweiger studied pharmacy at the University of Kiel. He received his PhD in the group of Prof. Christa Müller at the University of Bonn. Following a postdoctoral stay in her group and in the group of Prof. Jonathan Hall at ETH Zürich, he has headed a research group at TU Dortmund since 2013. His group develops DNA barcoding strategies for small‐molecule synthesis, designs encoded screening libraries, and uses encoded library technology to identify bioactive molecules*.